# Metabolomics Analysis of Urine Samples from Children after Acetaminophen Overdose

**DOI:** 10.3390/metabo7030046

**Published:** 2017-09-06

**Authors:** Laura K. Schnackenberg, Jinchun Sun, Sudeepa Bhattacharyya, Pritmohinder Gill, Laura P. James, Richard D. Beger

**Affiliations:** 1 Division of Systems Biology, National Center for Toxicological Research, US Food and Drug Administration, Jefferson, AR 72079, USA; jinchun.sun@fda.hhs.gov (J.S.); richard.beger@fda.hhs.gov (R.D.B.); 2 Arkansas Children’s Research Institute, Little Rock, AR 72202, USA; sbhattacharyya2@uams.edu (S.B.); PSGill@uams.edu (P.G.); jameslaurap@uams.edu (L.P.J.); 3 Department of Pediatrics, University of Arkansas for Medical Sciences, Little Rock, AR 72202, USA

**Keywords:** acetaminophen, biomarker, mass spectrometry, metabolomics, overdose

## Abstract

Acetaminophen (APAP), a commonly used over-the-counter analgesic, accounts for approximately fifty percent of the cases of acute liver failure (ALF) in the United States due to overdose, with over half of those unintentional. Current clinical approaches for assessing APAP overdose rely on identifying the precise time of overdose and quantitating acetaminophen alanine aminotransferase (ALT) levels in peripheral blood. Novel specific and sensitive biomarkers may provide additional information regarding patient status post overdose. Previous non-clinical metabolomics studies identified potential urinary biomarkers of APAP-induced hepatotoxicity and metabolites involved pathways of tricarboxylic acid cycle, ketone metabolism, and tryptophan metabolism. In this study, biomarkers identified in the previous non-clinical study were evaluated in urine samples collected from healthy subjects (*N* = 6, median age 14.08 years) and overdose patients (*N* = 13, median age 13.91 years) as part of an IRB-approved multicenter study of APAP toxicity in children. The clinical results identified metabolites from pathways previously noted, and pathway analysis indicated analogous pathways were significantly altered in both the rats and humans after APAP overdose. The results suggest a metabolomics approach may enable the discovery of specific, translational biomarkers of drug-induced hepatotoxicity that may aid in the assessment of patients.

## 1. Introduction

Acetaminophen (APAP) is a commonly used over-the-counter drug worldwide with a reported regular weekly consumption of 60 million by Americans [1]. While generally safe at the recommended dose, overdose of APAP accounts for half of the cases of acute liver failure (ALF) in the United States [2]. APAP is available in various formulations (tablets, liquids, suppositories, etc.) and in prescription and over-the-counter combination drugs; its widespread availability makes it more likely that a patient may consume more than the recommended dose. Approximately half the patients with APAP hepatotoxicity experience mild to moderate reactions, while approximately 48% are diagnosed with ALF; among those with ALF diagnoses, it has been reported that 29% require a liver transplant, with a 28% mortality rate [3]. Early therapeutic intervention with *N*-acetylcysteine (NAC) is critical to reduce the morbidity and mortality associated with APAP-induced hepatotoxicity. However, recognition of APAP hepatotoxicity can be challenging, as patients may be unaware of excessive exposure, and the early stages of toxicity are non-specific. Moreover, patients may present for medical evaluation after the time period for which NAC is most effective. ALT is often used to evaluate the severity of APAP-induced hepatotoxicity. However, there is a delay between ingestion of a toxic dose and the rise of ALT. Metabolomics-focused technologies can identify urinary biomarkers related to progression and recovery from APAP-induced hepatotoxicity. Such biomarkers, in conjunction with the clinical standards, may be useful in predicting how a patient will progress through the stages of APAP hepatotoxicity.

APAP-induced hepatotoxicity has been thoroughly investigated, and the mechanism is generally well understood. APAP is primarily metabolized by Phase II metabolic pathways to glucuronidated and sulfated conjugated forms, which are excreted in the urine. A small portion of APAP (~10% depending on amount ingested) undergoes metabolism to the highly reactive toxic metabolite *N*-acetyl-para-benzoquinone imine (NAPQI) that can bind to proteins and form APAP-protein adducts [4]. In addition, NAPQI can bind glutathione to form non-toxic cysteine and mercapturic acid conjugates, which are excreted in the urine. In the case of hepatotoxicity, the glucuronidation and sulfation pathways become saturated, resulting in the formation of excess NAPQI, which depletes the glutathione stores and leads to hepatocyte necrosis. APAP has been considered a model hepatotoxicant in metabolomics studies, and there have been multiple literature reports of the analysis of urine samples in animals [5,6,7,8,9,10,11,12,13,14,15,16] and humans following ingestion of APAP [5,10,17,18,19,20]. Many of the studies have reported changes in oxidative stress, glutathione-related and citrate cycle (TCA) metabolites, and altered fatty acids following ingestion of high doses of APAP.

The non-clinical studies can identify potential biomarkers of interest and provide pathway and mechanistic information. Importantly, it has been shown that the changes in the non-clinical models may be translated to the clinical setting. In this study, urine samples were collected from children or adolescents hospitalized with APAP overdose under an IRB-approved protocol [10,21,22]. Nuclear magnetic resonance (NMR)- and ultra-performance liquid chromatography/quadrupole-time-of-flight mass spectrometry (UPLC/QToF MS)-based metabolomics techniques were used to evaluate the urine samples from six control and 13 overdose patients. In particular, metabolites previously identified in the non-clinical APAP study [13] were evaluated in this study. Pathway analysis of both the clinical and non-clinical data was carried out and similar pathways were noted to be altered in both sample sets. The similarity between the clinical and the non-clinical data suggest that metabolomics can be used to identify translatable biomarkers and pathways, which may have relevance to the clinical assessment of patients with APAP hepatotoxicity.

## 2. Results

In this study, there were a total of six subjects in the control group and 13 subjects in the overdose group, which represented a subset of subjects from previous publications [10,22]. Table 1 provides the demographic and clinical chemistry data for the 19 subjects in the study.

Within the overdose group, three patients had urine samples collected at three or more timepoints, two patients had samples collected at two timepoints, and the remaining eight had urine samples from a single timepoint. For the control group, a single urine sample was acquired. The median age was 14.08 years for the control group, while the median age was 13.91 for the overdose subjects. Elevations in ALT for the control group were not noted; therefore, the peak ALT was not applicable. For the overdose group, the median peak ALT was 2050 IU/L, with values ranging from 106–6072 IU/L at the peak of ALT. The time to treatment for the APAP overdose group was variable, which may in part account for the large range in ALT values. Additionally, as noted, only five patients in the overdose group had samples collected over multiple timepoints, so the reported ALT value may not represent the maximum value in the patients with only one sample. APAP-protein adducts were measured in each group, with a reported median of 0.00 nmol/mL in the control group and a median level of 1.48 nmol/mL in the overdose group.

**Figure 1:**
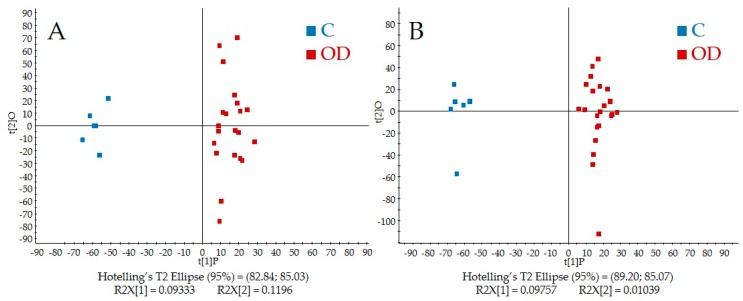
Partial least squares discriminant analysis (PLS-DA) scores plots for both (**A**) positive and (**B**) negative ionization modes. Control samples are shown in blue and samples from overdose subjects are in red.

Figure 1A,B shows the score plots from PLS-DA of the QToF-MS data in both positive and negative modes, respectively.

APAP and APAP metabolite peaks were removed prior to analysis, and Figure 1A,B shows a distinct separation between the control and overdose groups in both ionization modes. Closer examination of the scores plot reveals that urine samples from overdose patients collected at multiple timepoints clustered closely together. Individual metabolites from the QToF-MS spectra were evaluated based upon the loadings plot and identified based upon Human Metabolome Database or based upon authentic standards in an in-house database. Metabolites detected in the NMR spectra were quantified using Chenomx NMR Suite.

**Figure 2:**
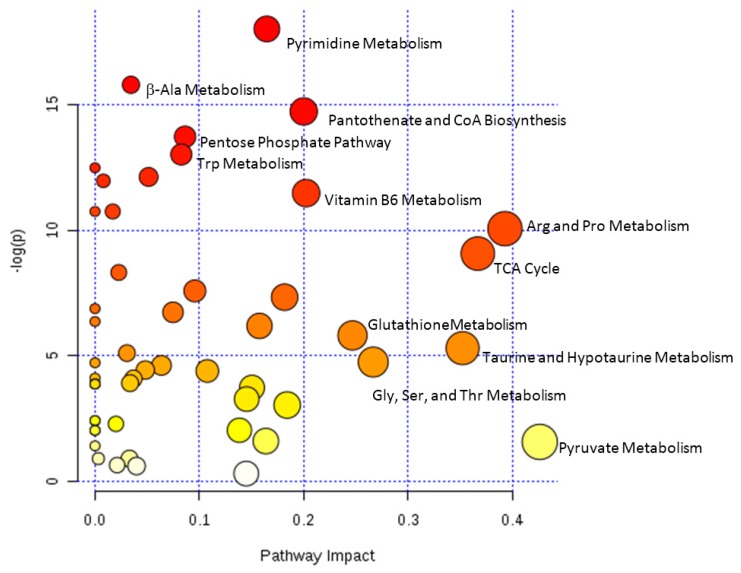
Metabolome view from pathway analysis performed using MetaboAnalyst. Select pathways with high pathway impact and/or high *p*-value are labeled.

The datasets from the identified metabolites from QToF-MS and NMR were combined for pathway analysis using MetaboAnalyst (Figure 2).

Significantly altered pathways (*p* < 0.05) that also had high impact values include arginine and proline metabolism, TCA cycle, taurine and hypotaurine metabolism, glycine, serine and threonine metabolism, and glutathione metabolism. Pathway significance is determined from pathway enrichment analysis and based upon the values for each compound in the dataset. The impact value, on the other hand, is determined by pathway topology analysis. Impact is determined based upon the importance of a metabolite within a pathway; a metabolite that is found at a junction point within a pathway may have a greater impact on the pathway function if the level is altered. Pyruvate metabolism had a high impact, although the *p*-value was not significant. The impact value for pyruvate metabolism was high, since pyruvate, lactate, and acetate are all located at junctions within the pathway (Figure S9); however, the changes in these metabolites were not significant in control versus overdose, resulting in a non-significant *p*-value for the pathway. Other pathways that were significantly altered but with lower impact included tryptophan, pyrimidine, vitamin B6, and b-alanine metabolism; pantothenate and CoA biosynthesis; and the pentose phosphate pathway. Among the high impact pathways, pyruvate metabolism, glycine, serine, and threonine metabolism, and arginine and proline metabolism are all connected to the citric acid cycle. Furthermore, taurine and hypotaurine metabolism as well as arginine and proline metabolism are connected to glutathione metabolism, which was also significantly altered. The results are consistent with our previous study of APAP-induced hepatotoxicity in Sprague Dawley rats, which indicated altered energy and glutathione pathways [13].

Metabolites from NMR or QToF-MS with a significant correlation to ALT or APAP-protein adducts are presented in Table 2.

Among the reported metabolites, many are related to the significantly altered pathways identified in the pathway analysis. In addition, taurocholic acid had a significant correlation to APAP-protein adducts. Taurocholic acid and other bile acids have previously been shown to be altered due to APAP-induced hepatotoxicity in both the rodent model and clinical samples [5,21,22]. Hydroxybutyrylcarnitine had a significant negative correlation to ALT levels. The acylcarnitines have also been shown to be altered early in APAP-induced hepatotoxicity in the rodent model [5,13], with the maximum in the acylcarnitine occurring prior to the maximum of ALT. *p*-Values were adjusted for multiple comparison testing using the false discovery rate (FDR) and after adjustment; only the correlations for hippurate, ascorbic acid, and propylene glycol were significant.

## 3. Discussion

Hepatotoxicity due to APAP overdose remains a critical issue, with high rates of morbidity and mortality that can occur if not recognized quickly [1,23,24,25]. In this study, metabolomics analyses have been employed to evaluate clinical urinary biomarkers of APAP-induced hepatotoxicity. Such non-invasive biomarkers may be useful in determining the stage of hepatotoxicity in the clinical setting and may predict prognosis when used in conjunction with the current clinical measures.

The urinary metabolites reported in Table 2 represent several different pathways that have been previously identified in non-clinical models of APAP-induced hepatotoxicity, including glutathione metabolism, bile acids metabolism, the citric acid (TCA) cycle, and fatty acid β-oxidation [6,14,15,21,26,27,28]. Furthermore, a previous clinical study evaluated the ability of urine metabolites to predict APAP-induced toxicity in humans identified responders versus nonresponders based upon a subset of metabolites [29]. Among the metabolites reported in the study by Winnike and coworkers [29], alanine, hippurate, creatinine, and trimethyl amine-*N*-oxide were also found to be correlated with ALT or APAP protein adducts, as shown in Table 2. Alanine and hippurate were increased in the overdose group in this study and in the group of responders from the study by Winnike et al. [29], while creatinine was decreased in the overdose group and the responders.

The relatively increased formation of toxic NAPQI results in depletion of glutathione (GSH). Glutathione is important in the detoxification of reactive molecules, and depletion of GSH following APAP overdose has been well-established. While GSH was not measured directly in this study, pathway analysis showed that the glutathione metabolism pathway was significantly altered with a high impact value. In addition, multiple pathways associated with glutathione metabolism were also significantly altered, including taurine and hypotaurine metabolism, arginine and proline metabolism, and glycine, serine, and threonine metabolism. These alterations in glutathione metabolism and other glutathione-relevant pathways may reflect an effort at the cellular level to replenish GSH stores to detoxify NAPQI and prevent further liver injury.

Previous reports [5,6,13,30] have noted that APAP overdose can result in global energy failure potentially through the inhibition of fatty acid β-oxidation. This study also showed that the TCA cycle was altered in the overdose group compared to the control group. Pathway analysis indicated that the TCA cycle had a high impact value and significant *p*-value (*p* < 0.05). In addition, Table 2 shows that hydroxybutyrylcarnitine had a significant negative correlation with ALT. It was previously reported that the acylcarnitines were elevated in serum from rats given a toxic dose of APAP prior to an increase in ALT levels [13]. Acylcarnitines have been proposed as blood-based biomarkers of reduced β-oxidation and mitochondrial dysfunction. In healthy individuals, urinary acylcarnitine concentrations are typically negligible [31,32]. It has been hypothesized that the alteration in fatty acid β-oxidation may reflect a shift in energy metabolism to glycolysis to make up for the loss of ATP. Further studies are needed to confirm the switch to glycolysis in the clinical samples.

In this study, the time from subject’s overdose to presentation in the hospital was variable, along with the time to NAC treatment. Furthermore, the study population represents a small cohort with six control subjects and 13 overdose patients, and more studies are needed to verify the results and determine whether other factors like genetics play a role in the response to acetaminophen. However, despite these challenges, the results from this clinical metabolomics study were consistent with previous findings in a rat model of APAP-induced hepatotoxicity. Metabolites from arginine and proline metabolism, TCA cycle, taurine and hypotaurine metabolism, glycine, serine and threonine metabolism, and glutathione metabolism pathways were significant in the APAP overdose group compared with the control group.

## 4. Materials and Methods

### 4.1. Ethical Approval

As part of a multicenter study on the toxicity of APAP in children ages 2–18, biological samples including urine were collected from subjects. The study was approved by the institutional review boards of all participating institutions, in accordance with the guidelines of the 1975 Declaration of Helsinki. In this study, only a subset of the urine samples was evaluated that fell directly under the purview of the University of Arkansas for Medical Sciences (UAMS) Institutional Review Board. Informed consent (and assent) documents were approved by the UAMS Institutional Review Board.

### 4.2. Subjects and Sample Collection

Complete details on the subjects and sample collection can be found in James et al. [10]. Urine samples from the control group and the overdose group were evaluated in this study. The control group (*N* = 6) included healthy subjects with no APAP use in the preceding 14 days, while the overdose group (*N* = 13) included children or adolescents hospitalized with APAP overdose as determined by published guidelines. The control group had a single urine sample collected, while the overdose group had multiple timepoints collected during hospitalization. Additionally, clinical and demographic data were collected and stored in a database that also included ALT levels.

### 4.3. Metabolomics Methods

#### 4.3.1. Chemicals

Optima LC/MS grade acetonitrile and water were purchased through Fisher Scientific (Pittsburgh, PA, USA). Formic acid and leucine-enkephalin were purchased from Sigma-Aldrich (St. Louis, MO, USA).

#### 4.3.2. UPLC/QToF-MS Analysis

A 5 μL volume of urine diluted by water (1:10) was injected on to a Waters Acquity Ultra Performance Liquid Chromatography (UPLC) system equipped with a Waters bridged ethyl hybrid (BEH) C18 column (2.1 mm × 10 cm; 1.7 μm particle size) held at 40 °C for separation of the metabolites. In addition to the individual urine samples, a pooled sample was prepared from an aliquot of each sample and run along with blank samples at regular intervals during the sample analysis. The UPLC mobile phases were 0.1% formic acid in water (solution A) and 0.1% formic acid in acetonitrile (solution B). Metabolites were eluted at a constant flow rate of 0.4 mL/min over the following gradient: 0–30% B from 0 to 6 min, 30–50% B from 6 to 9 min, and 50–95% B from 9–11 min. The final gradient composition was held for 1 min and returned to 100% A at 12.1 min. Following elution from the column, metabolites were detected with a QToF Premier mass spectrometer (Waters, Milford, MA, USA) operated in positive and negative ionization electrospray modes. Capillary voltages were 3.2 kV and 2.4 kV for positive and negative modes, respectively, with a cone voltage of 40 V in both modes. Data was collected in full scan mode from *m*/*z* 100 to 900 in both ionization modes. In order to assure mass accuracy, leucine-enkephalin at a concentration of 250 pg/μL (in 50:50 acetonitrile:0.1% formic acid) was used as the lock-mass in positive mode ([M + H]^+^ = 556.2771) and at a concentration of 25 ng/μL for negative mode ([M − H]^−^ = 554.2615).

Micromass MarkerLynx XS Application Version 4.1 (Waters, Milford, MA, USA) with extended statistical tools was employed for peak selection and peak alignment of the raw data. Peaks were extracted using the parameter settings: XIC window 0.02 Da; retention time window 0.02 min; peak width at 5% height 15S; peak-to-peak baseline noise 50. Prior to statistical analysis, the data was normalized based upon total ion intensity. The resulting aligned dataset was analyzed by unsupervised principal component analysis (PCA) and supervised partial least squares discriminant analysis (PLS-DA). The resulting ion intensities and retention times were exported as EXCEL files. The majority (>90%) of the metabolites detected by MS were identified based on the accurate mass measurement and retention time by comparison with an in-house database, while the rest (<10%) were putatively identified by accurate mass (<0.02 Da or 6 ppm) and comparison of the fragment mass spectra with the fragment mass spectra in the Human Metabolome Database (HMDB; www.hmdb.ca). Supplementary Figure S19 indicates how unknowns were identified based upon comparison of the fragmentation mass spectrum to the fragmentation mass spectrum of a standard compound in an in-house database.

#### 4.3.3. Nuclear Magnetic Resonance Spectroscopy Analysis

Urine samples were combined with 4,4-dimethyl-4-silapentane-1-sulfonic acid (DSS, chemical shift standard) in D_2_O, difluoro trimethylsilanyl phosphonic acid (DFTMP, pH standard), and sodium phosphate buffer for analysis. A pooled urine sample was also prepared for NMR and run at regular intervals during data analysis. Urine samples were analyzed on a Bruker Avance NMR spectrometer operating at 600.133 MHz for proton and equipped with a Bruker QNP cryoprobe. Water suppression was achieved using the noesy1dpr pulse sequence. For each sample, 128 scans were collected into 32 K data points over a spectral width of 9615.39 Hz. Raw data was processed in ACD/Labs 1D NMR Manager (ACD/Labs, Toronto, ON, Canada). Processed data were grouped and integrated. The individual spectra exported were as *.jdx files for quantification of select metabolites in Chenomx NMR Suite (Edmonton, AB, Canada). The metabolite concentrations were normalized to the urine creatinine concentration in each sample.

#### 4.3.4. Pathway Analysis

MetaboAnalyst 3.0 was employed for pathway analysis of the clinical and nonclinical data [33,34,35,36,37,38,39]. The NMR and MS data were combined and the HMDB ID used as the compound label. The dataset comprised a total of 118 unique compounds from the NMR and MS platforms for pathway analysis. Once matched, the data was mean-centered and divided by the standard deviation of each variable. The Homo sapiens pathway library was selected for the analysis with the global test selected as the pathway enrichment analysis method. For pathway topology analysis, relative betweenness centrality was selected to estimate node importance. The output from the analysis included metabolome (Figure 2), pathway and compound views to explore the data; KEGG pathways with key metabolites highlighted and compound views are provided in Supplementary material (Figures S1–S17). In the report, pathway significance is determined from pathway enrichment analysis and based upon the values for each compound in the dataset. The impact value, on the other hand, is determined by pathway topology analysis and the importance of each pathway calculated based upon the cumulative percentage from matched metabolite nodes.

#### 4.3.5. Correlations Analysis

The Pearson correlations between ALT or protein adducts were calculated in R using the rcorr function within the Hmisc package. Both the correlation coefficient and the *p*-value for the correlation were returned from the calculation. The FDR-adjusted *p*-values were calculated in R using the Benjamini & Hochberg correction [40].

## Figures and Tables

**Table 1:** Demographics and clinical data for control and overdose subjects. Peak acetaminophen alanine aminotransferase (ALT) and adducts are reported as the median (range).

Variables	Control *N* = 6	Overdose *N* = 13
Age (years)	14.08	13.91
PEAK ALT (IU/L)	NA	2050 (106, 6072)
Peak Adduct (nmol/mL)	0.00 (0.00, 0.00)	1.48 (0.20, 6.69)

**Table 2:** Select metabolites with high correlations to alanine aminotransferase (ALT) or acetaminophen (APAP)-protein adducts. Metabolites with significant correlations (*p* < 0.05) to ALT or APAP-protein adducts are in bold.

Metabolite	Platform	ALT	APAP-Protein Adducts
2-Oxoarginine ^a^	LCMS POS, 0.72 min, *m*/*z* 174.09	0.6192, *p =* 2.40 × 10^−2^	0.4318
Ascorbic acid ^a^	LCMS POS, 0.72 min, *m*/*z* 177.04	0.4361	0.8446, *p =* 2.81 × 10^−4^
Ascorbic acid ^a^	LCMS NEG, 0.72 min, *m*/*z* 175.02	0.3889	0.8159, *p =* 6.72 × 10^−4^
Alanine	NMR, 1.47, 3.78 ppm	0.2600	0.6792, *p =* 1.073 × 10^−2^
Choline	NMR, 3.19, 3.51, 4.06 ppm	0.1789	0.5993, *p =* 3.04 × 10^−2^
Citrulline ^a^	LCMS POS, 0.67 min, *m*/*z* 176.10	−0.3810	−0.6676, *p =* 1.27 × 10^−*2*^
Cresol ^a^	LCMS NEG, 3.77 min, *m*/*z* 107.05	−0.5642, *p =* 4.46 × 10^−2^	−0.4348
Fructose	NMR, 3.55–4.11 ppm	0.3900	0.8170, *p =* 6.51 × 10^−4^
Glucose	NMR, 3.23–3.89, 4.64 ppm	0.2778	0.6367, *p* = 1.93 × 10^−*2*^
Hippurate ^a^	LCMS NEG, 3.32 min, *m*/*z* 178.05	0.4858	0.8561, *p =* 1.88 × 10^−4^
Hippurate	NMR, 3.96, 7.54, 7.63, 7.82 ppm	0.4116	0.8350, *p =* 3.82 × 10^−4^
Hydroxybutyrylcarnitine ^a^	LCMS POS, 1.05 min, *m*/*z* 248.15	−0.5674, *p =* 4.46 × 10^−2^	−0.4900
Indoxyl ^a^	LCMS POS, 3.30 min, *m*/*z* 134.06	0.7062, *p =* 6.98 × 10^−3^	0.8230, *p =* 5.49 × 10 ^−4^
Lactate	NMR, 1.32, 4.11 ppm	-	0.7906, *p =* 1.29 × 10^−3^
Proline ^a^	LCMS POS, 0.83 min, *m*/*z* 116.07	−0.5733, *p =* 4.05 × 10^−2^	−0.5376
Propylene glycol	NMR, 1.13, 3.44, 3.54, 3.88 ppm	0.4025	0.8263, *p =* 4.99 × 10^−4^
Pyruvate	NMR, 2.36 ppm	0.3680	0.7134, *p* = 6.18 × 10^−3^
Taurocholic acid isomer ^b^	LCMS NEG, 4.83 min, *m*/*z* 514.28	0.4259	0.6891, *p* = 9.18 × 10^−3^
Trimethylamine N-oxide	NMR, 3.25 ppm	0.5807, *p* = 3.74 × 10^−2^	0.3733
Uracil ^a^	LCMS POS, 0.78 min, *m*/*z* 113.03	-	0.6349, *p =* 1.97 × 10^−2^
Uric acid ^a^	LCMS NEG, 0.83 min, *m*/*z* 167.02	-	0.5636, *p =* 4.49 × 10^−2^

^a^ Compounds identified based upon an authentic standard from an in-house database; ^b^ Compounds identified from the Human Metabolome Database.

## Supplementary Materials

The following are available online at www.mdpi.com/2218-1989/7/3/46/s1: Figures S1–S19. Figures S1–S12 show the KEGG pathways for pathways with high impact and/or high *p*-value from MetaboAnalyst pathway analysis with key metabolites from pathway analysis highlighted by red circles. Figures S13–S17 show boxplots for select metabolites of interest as determined by pathway analysis. Figure S18 shows the PCA plots for LC/MS analysis in both positive and negative ionization modes. Figure S19 shows the process for identifying an unknown ion in the LC/MS data based upon comparison to an authentic standard.
