# Supplementary material for: Metabolomics Analysis of Urine Samples from Children after Acetaminophen Overdose
Source: Metabolites. 2017 Sep 6;7(3):46. doi: 10.3390/metabo7030046 (PMC5618331; doi:10.3390/metabo7030046)
Supplement: Supplementary file 1 [file metabolites-07-00046-s001.pdf]

# Supplementary Materials: Metabolomics Analysis of Urine Samples from Children after Acetaminophen-Overdose

**Laura K. Schnackenberg, Jinchun Sun, Sudeepa Bhattacharrya, Pritmohinder Gill, Laura P. James, and Richard D. Beger**

## 1. KEGG Metabolic Pathways with High Impact and/or High Significance in Figure 2

KEGG metabolic pathways with high impact and/or high p value determined from the MetaboAnalyst pathway analysis tool with matched metabolites highlighted with red circles. The metabolites were matched from pathway analysis using the uploaded data from MS and NMR analyses.

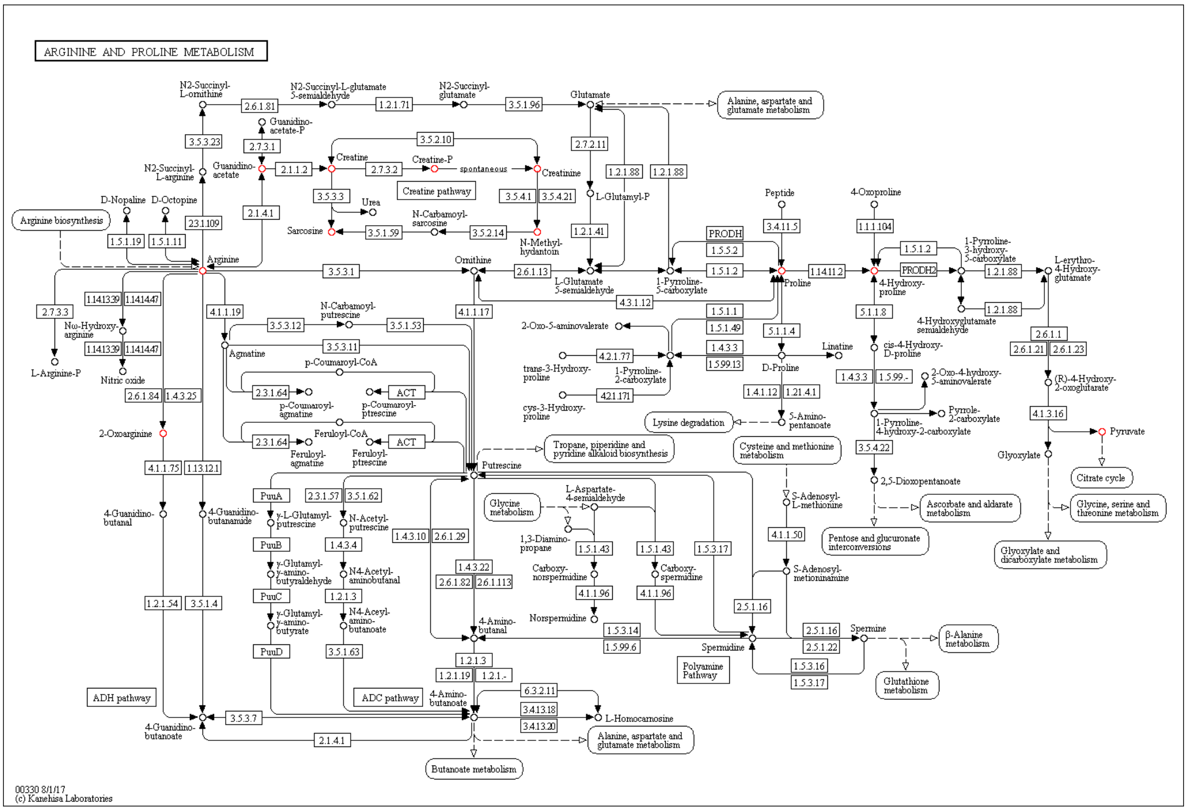


**Figure S1.** Arginine and proline metabolism.

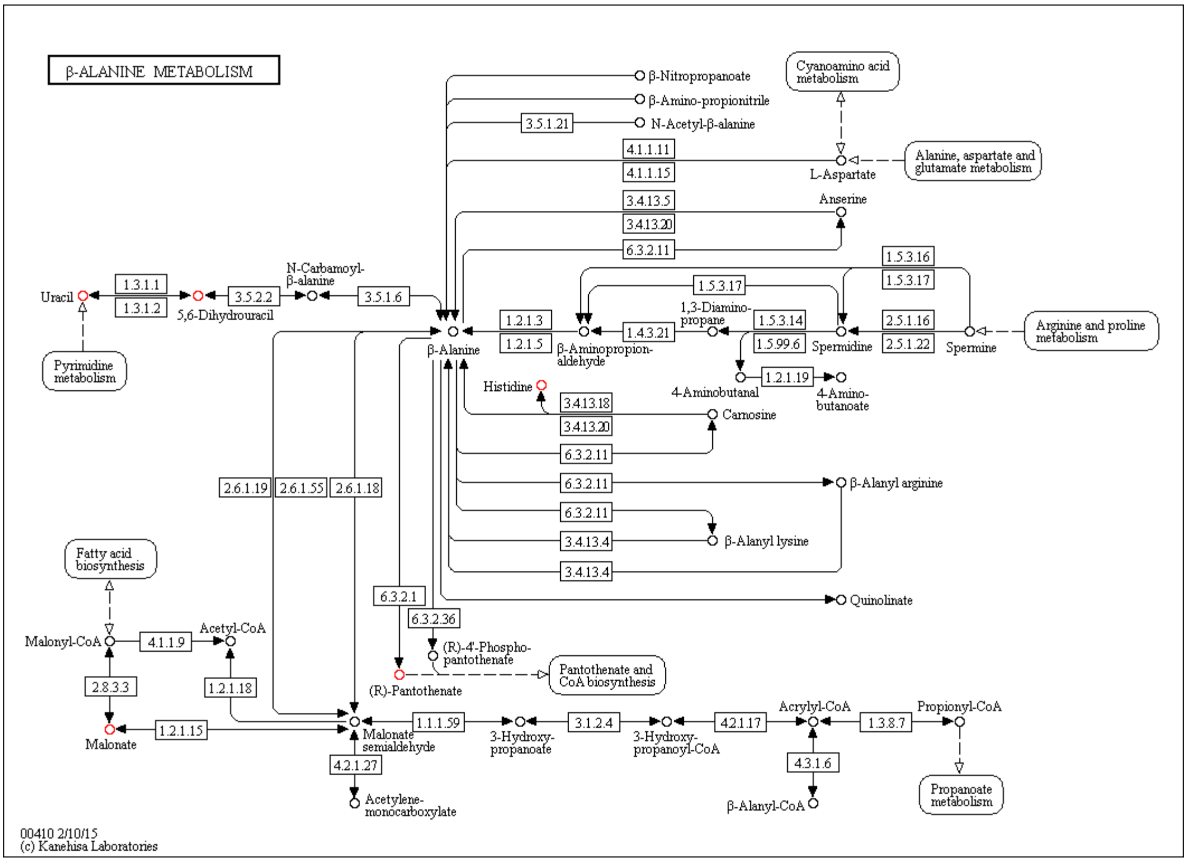


**Figure S2.** β-alanine metabolism.

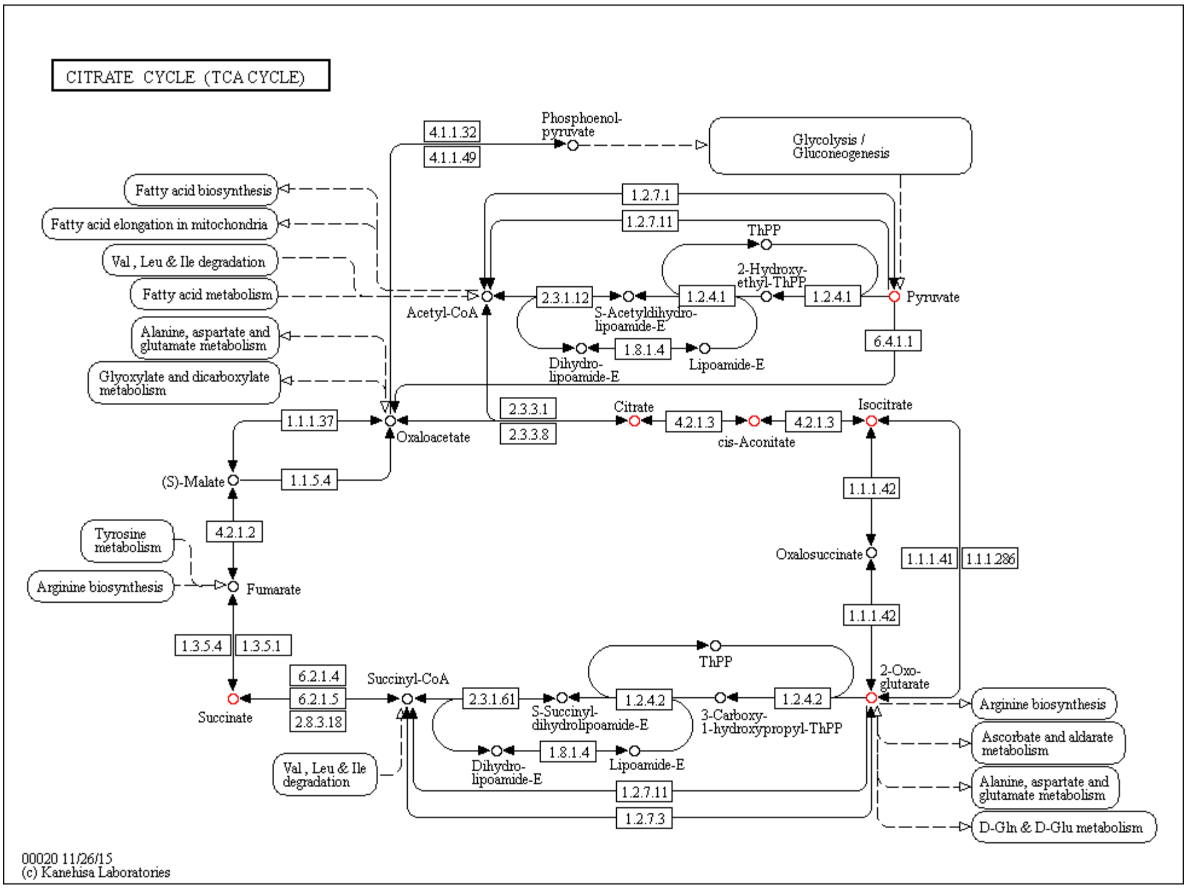


**Figure S3.** Citrate cycle (TCA cycle).

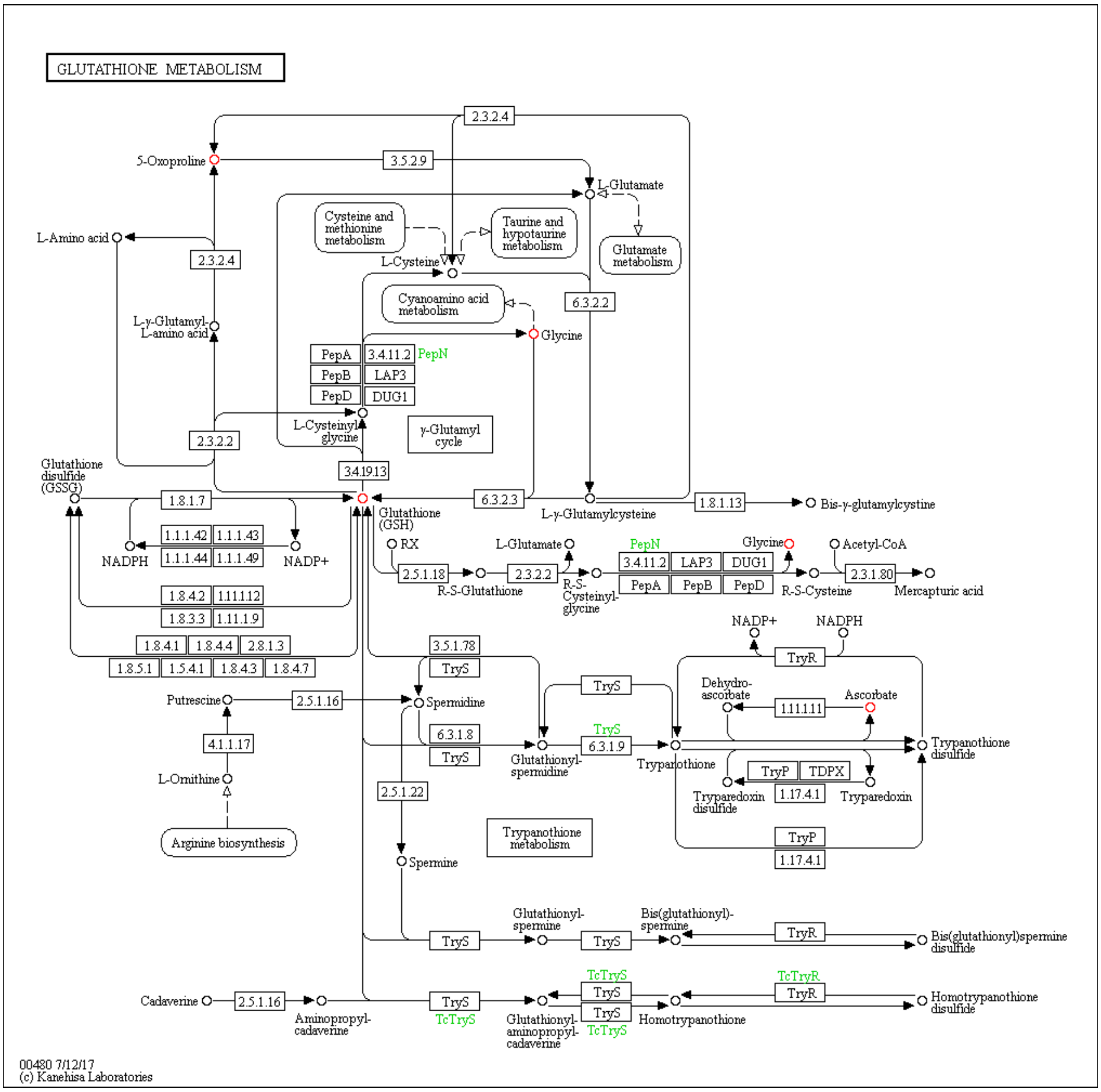


**Figure S4.** Glutathione metabolism.

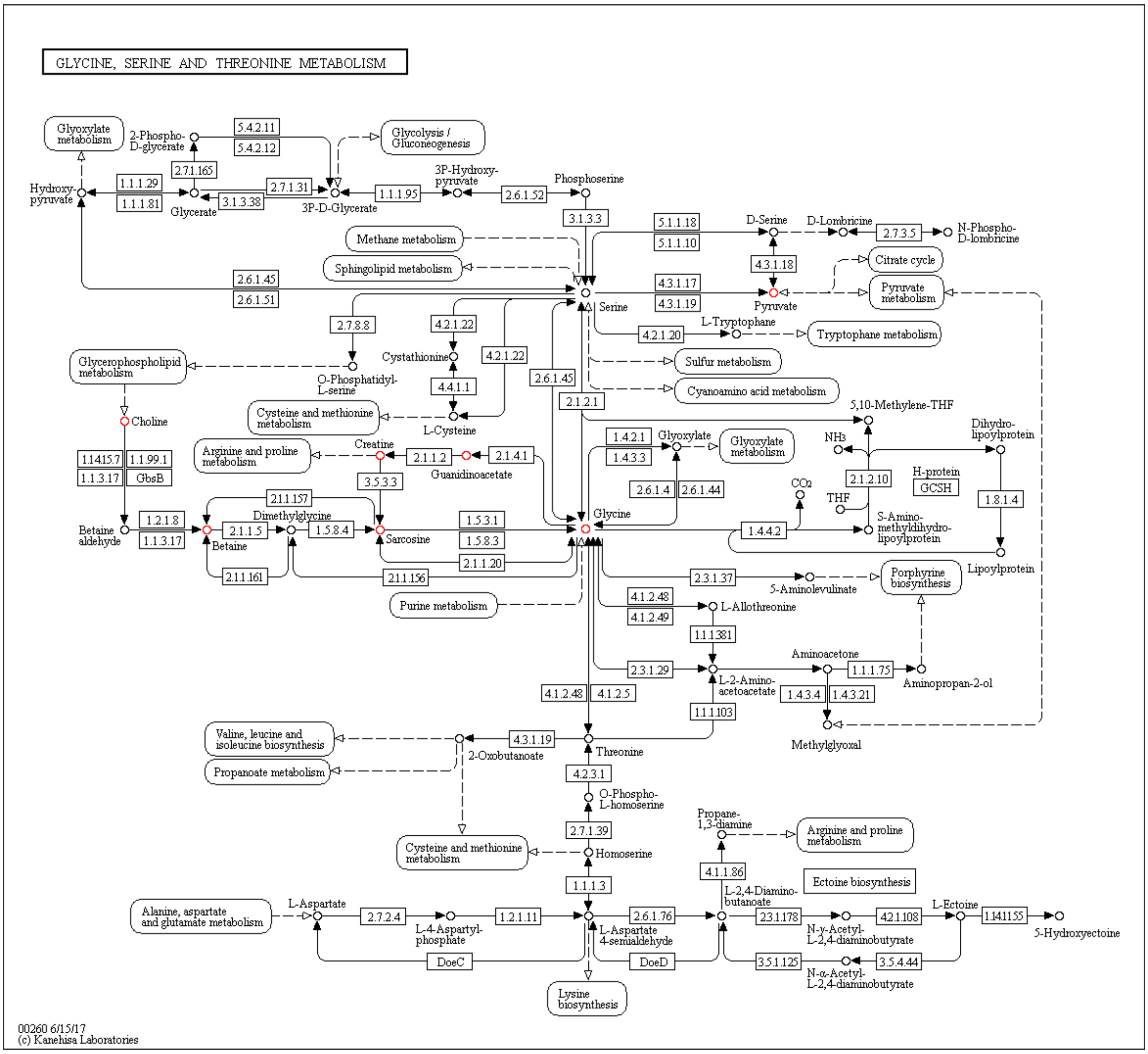


**Figure S5.** Glycine, serine, and threonine metabolism.

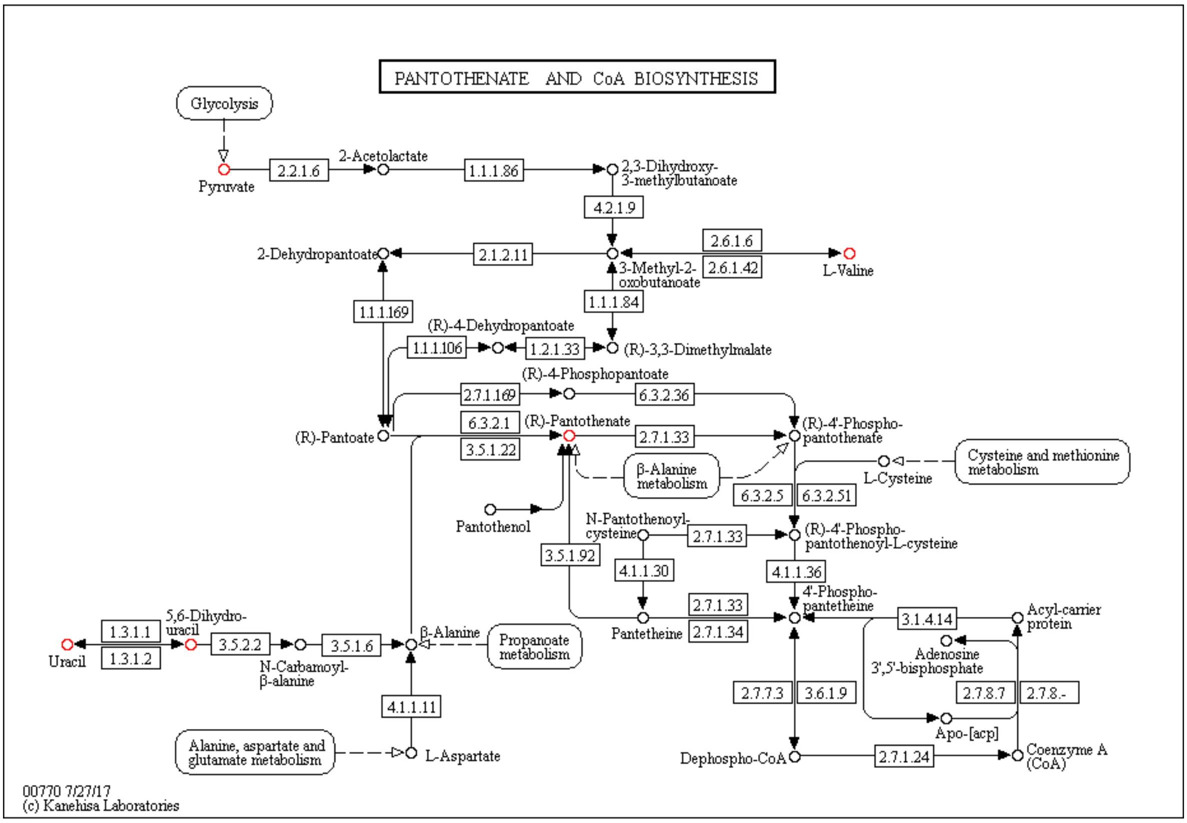


**Figure S6.** Pantothenate and CoA biosynthesis.

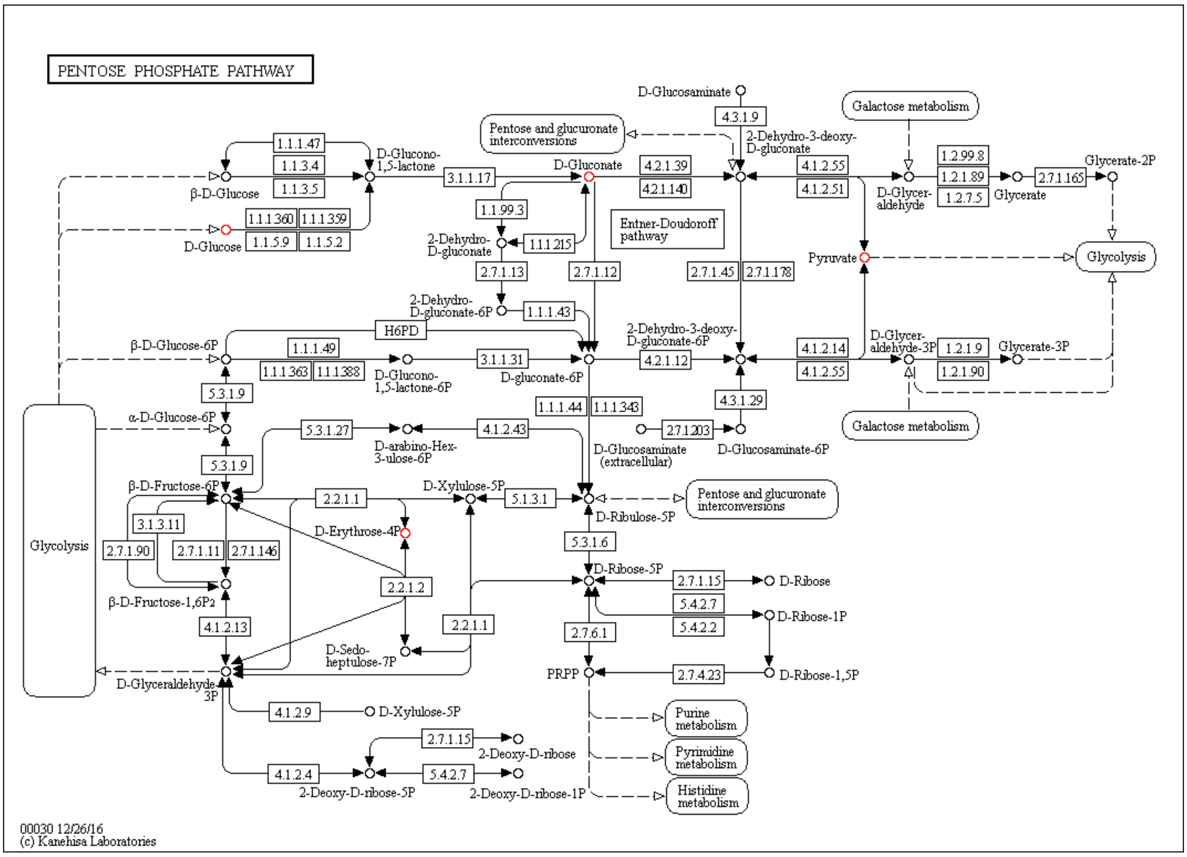


**Figure S7.** Pentose phosphate pathway.

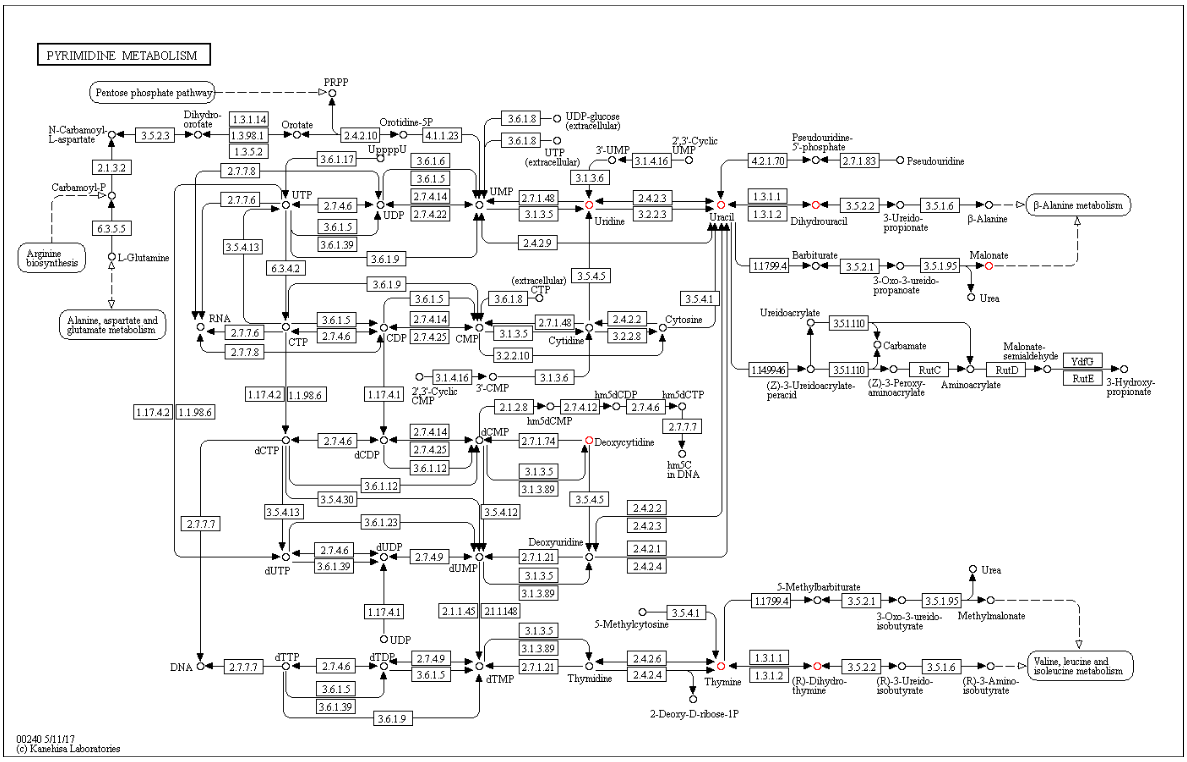


**Figure S8.** Pyrimidine metabolism.

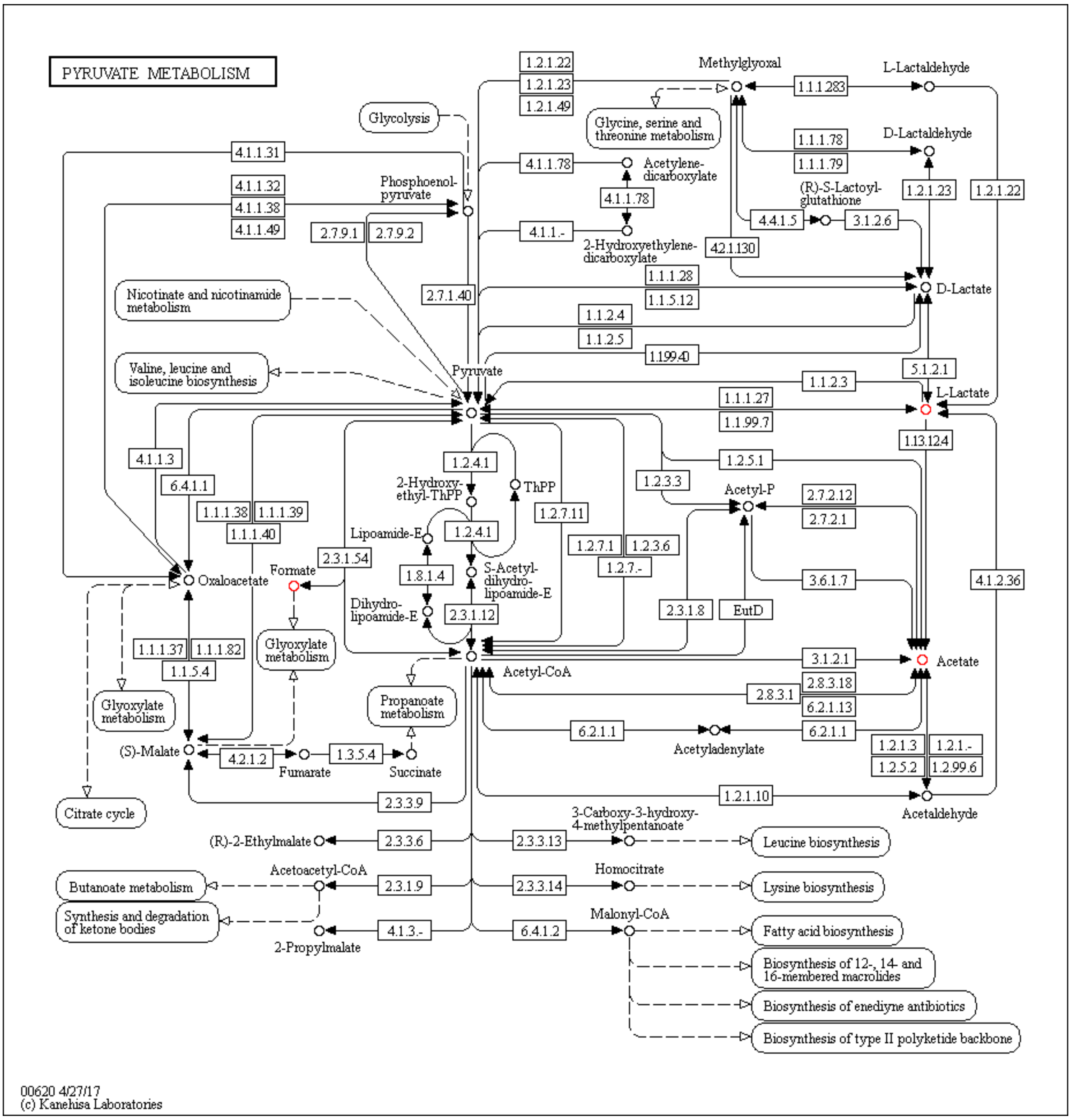


**Figure S9.** Pyruvate metabolism.

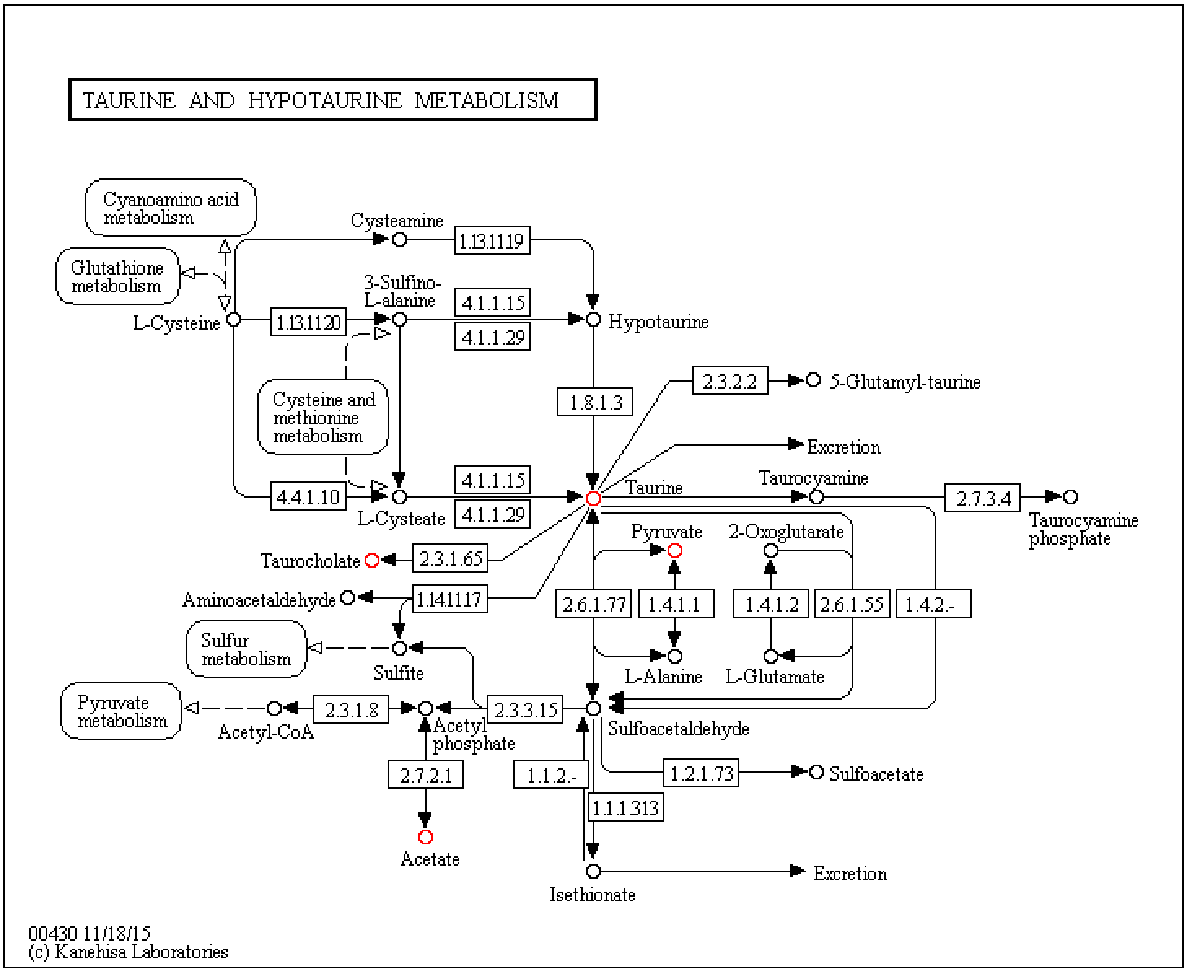


**Figure S10.** Taurine and hypotaurine metabolism.

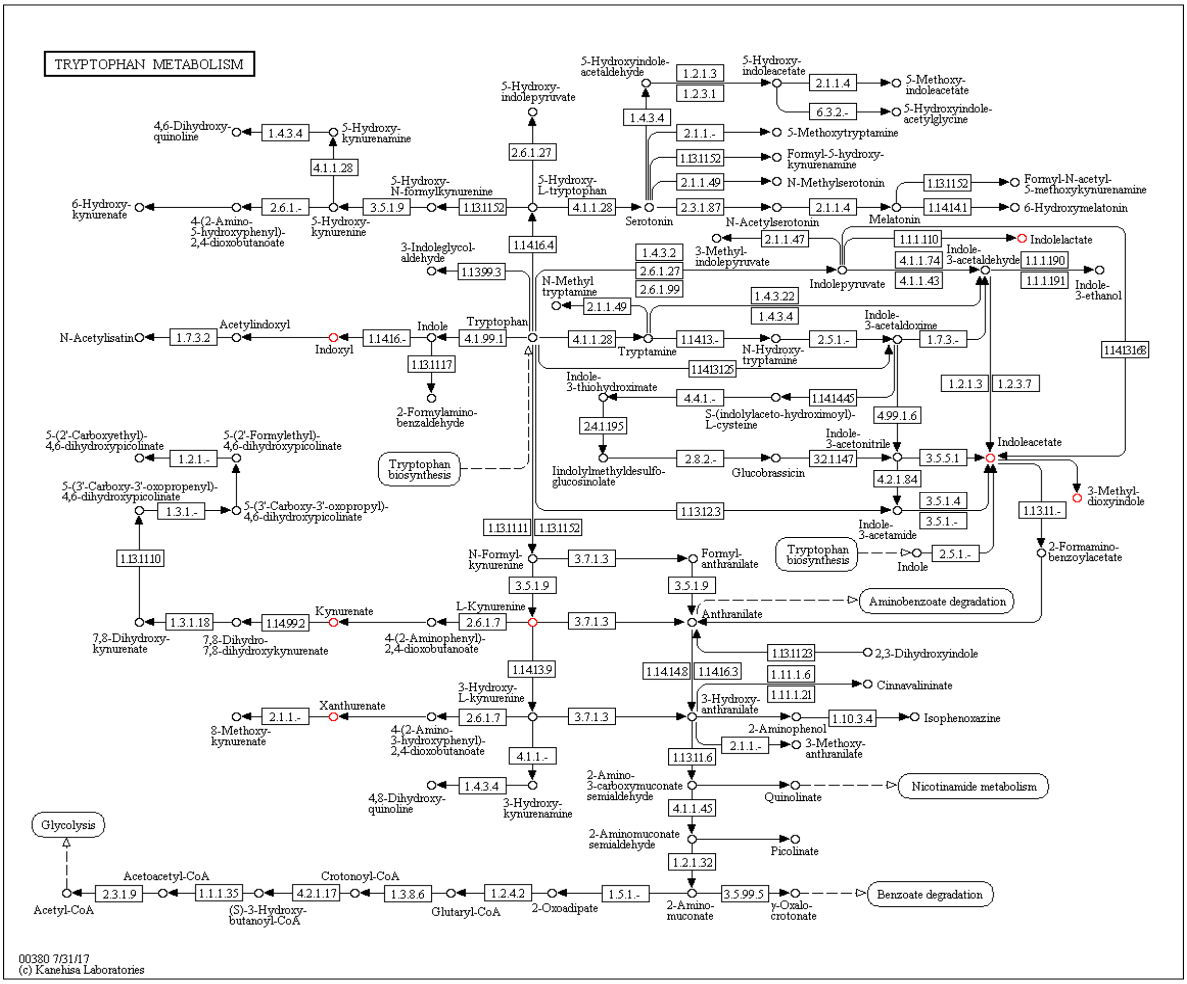


**Figure S11.** Tryptophan metabolism.

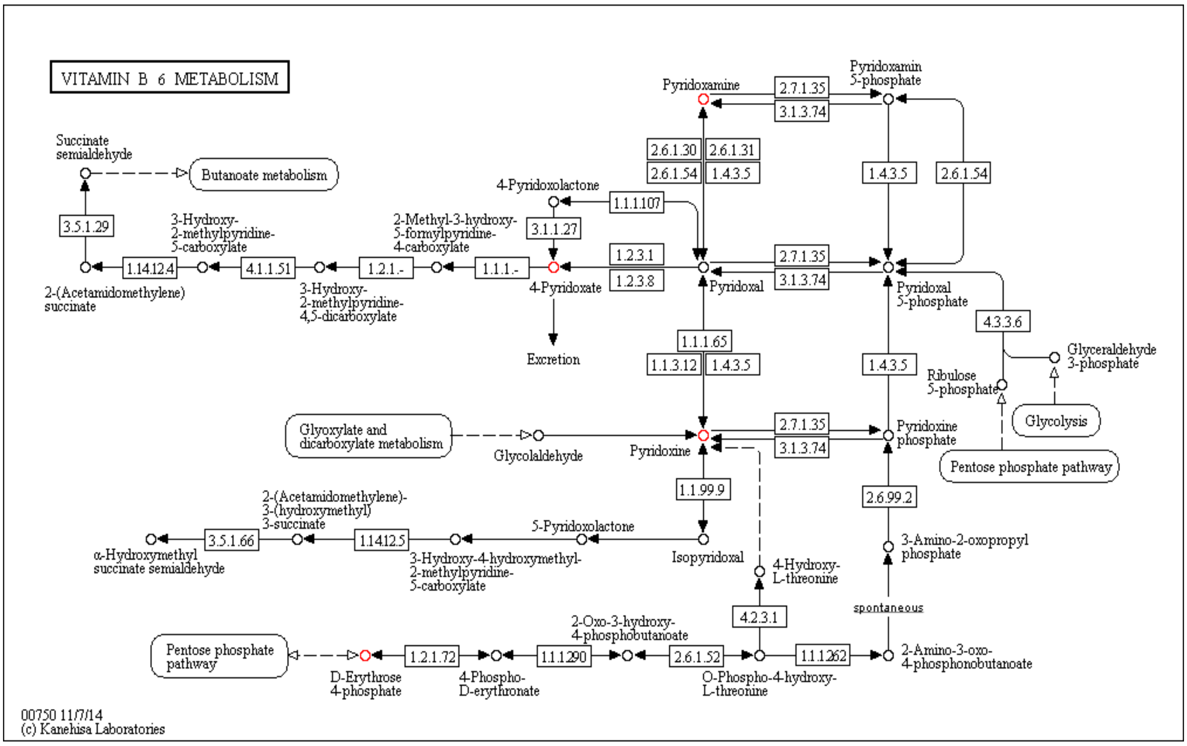


**Figure S12.** Vitamin B6 metabolism.

## 2. Compound Views

Compound views from MetaboAnalyst pathway analysis for select metabolites. The boxplots provide distributions of compounds with regard to phenotype label, in this case, control versus overdose.

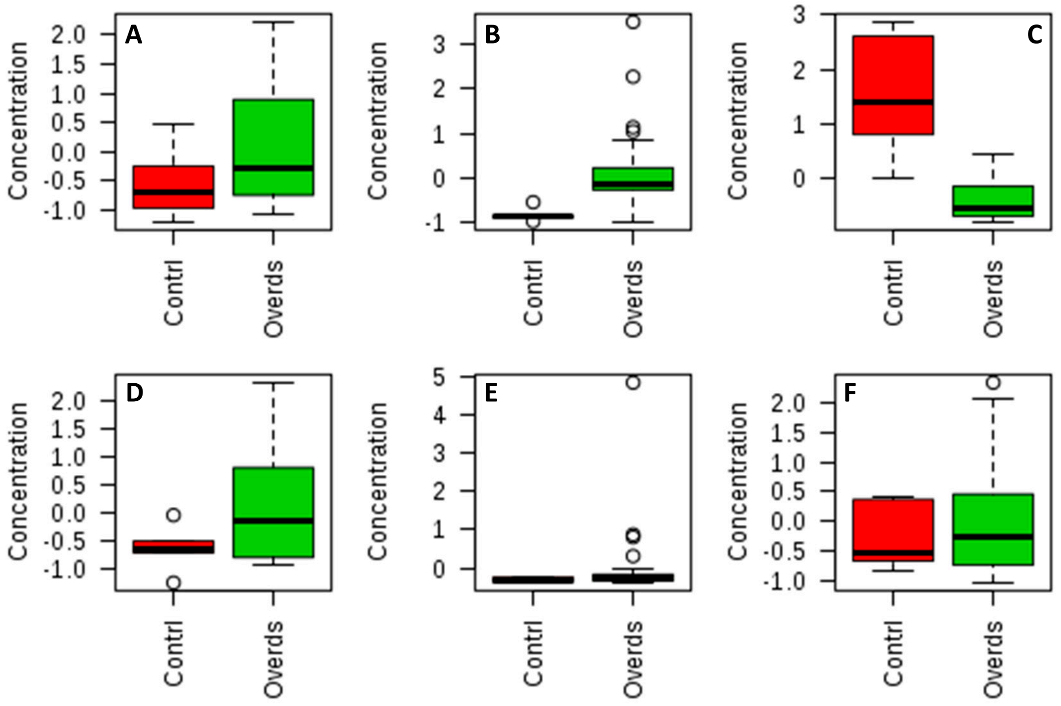


**Figure S13.** Pathway Views for a) hydroxyphenyllactic acid, b) pyruvic acid, c) D-glucose, d) glycine, e) acetic acid, and f) succinic acid.

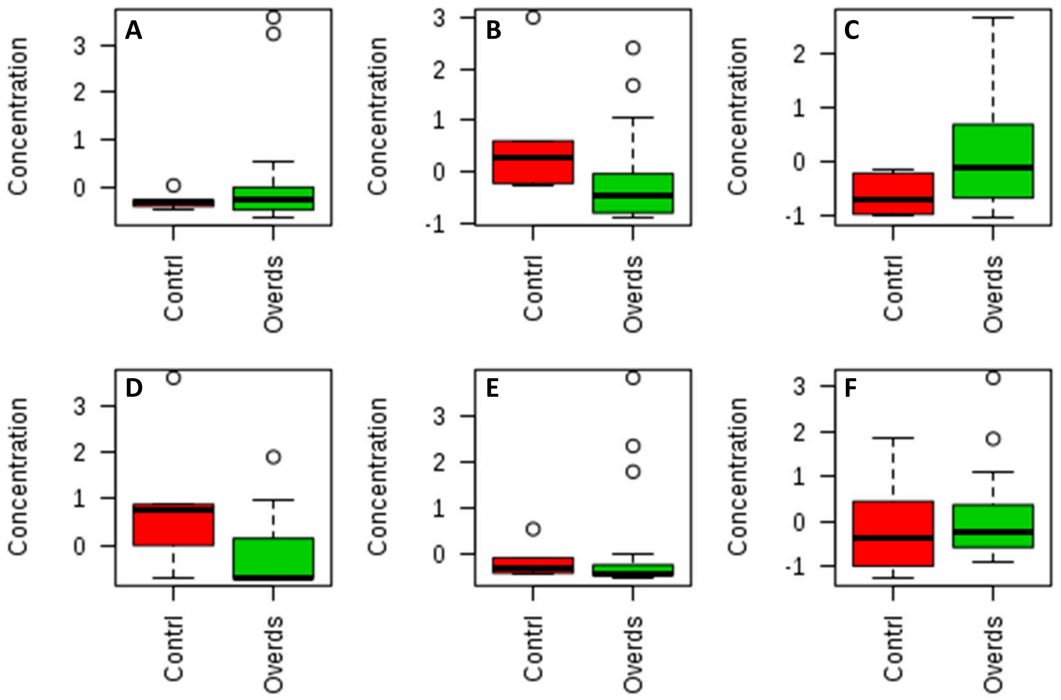

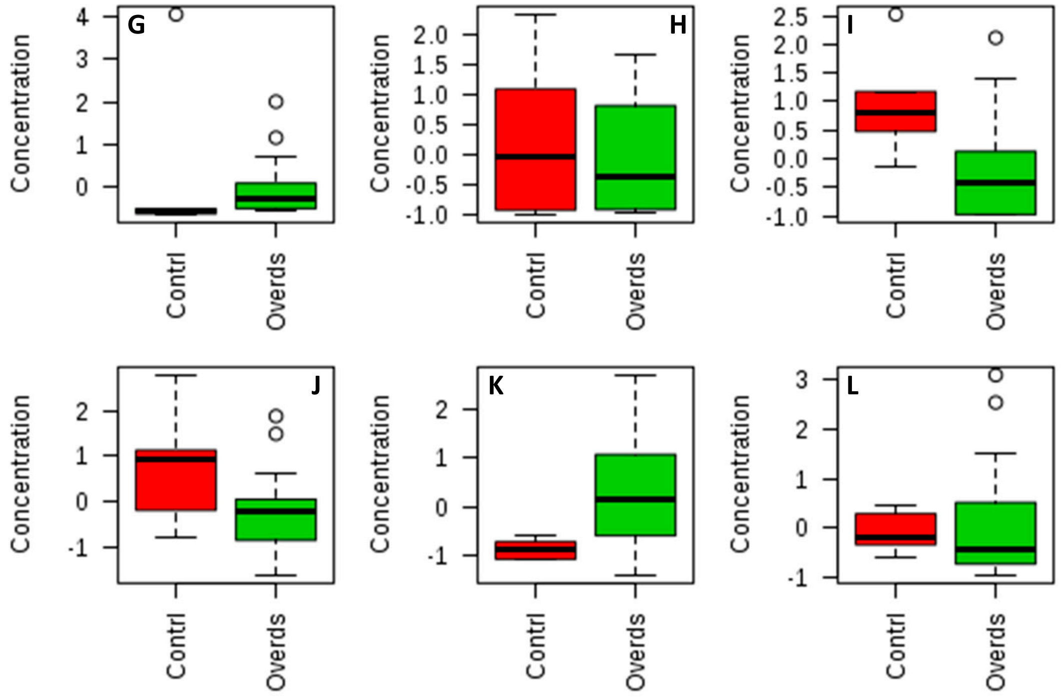


**Figure S14.** Pathway Views for a) formic acid, b) glutathione, c) L-arginine, d) uracil, e) ascorbic acid, f) choline, g) thymine, h) glycolic acid, i) citric acid, j) L-proline, k) L-histidine, and l) L-valine.

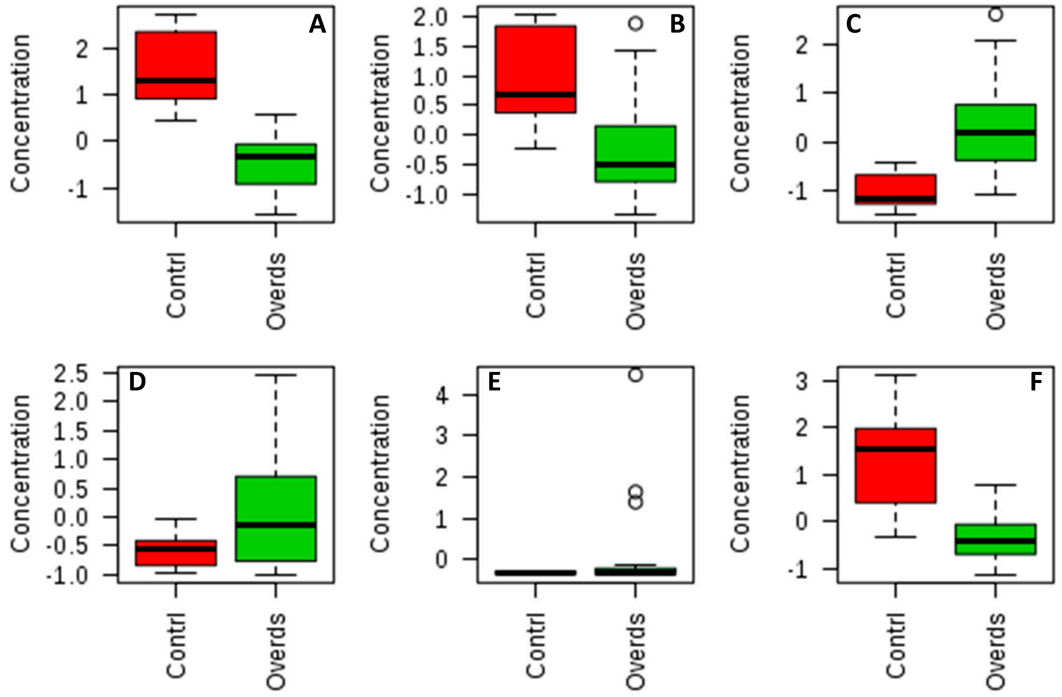


G I

 H

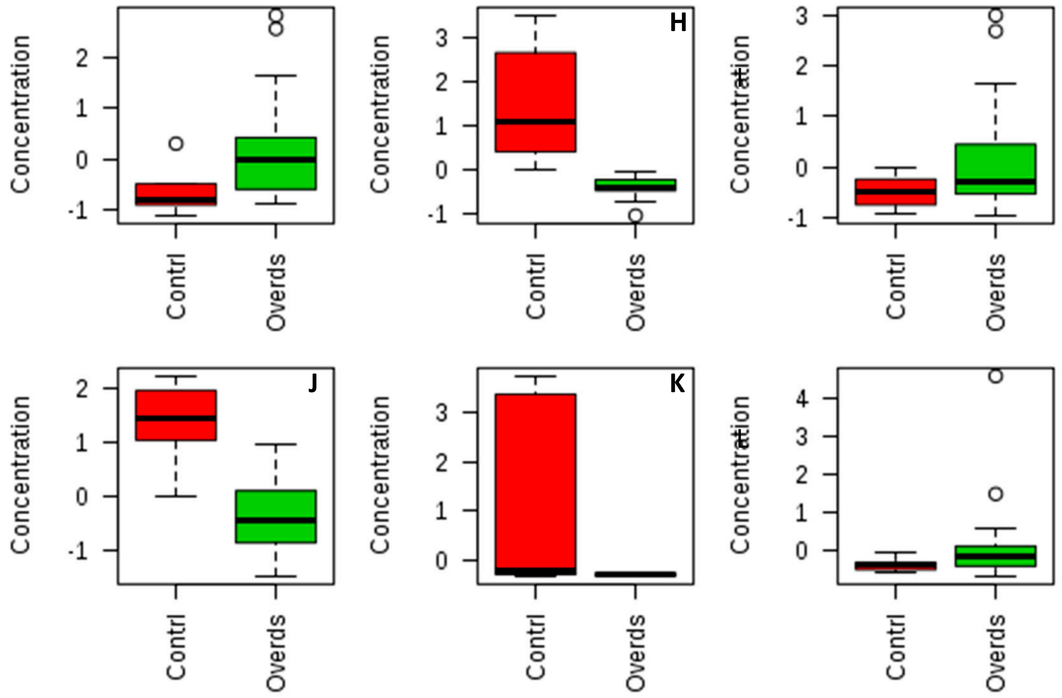


**Figure S15.** Pathway Views for a) D-erythrose 4-phosphase, b) gluconic acid, c) sulfolithocholylglycine, d) sarcosine, e) L-lactic acid, f) uridine, g) L-kynurenine, h) citrulline, i) pyroxidine, j) isocitric acid, k) creatine, and l) malonic acid.

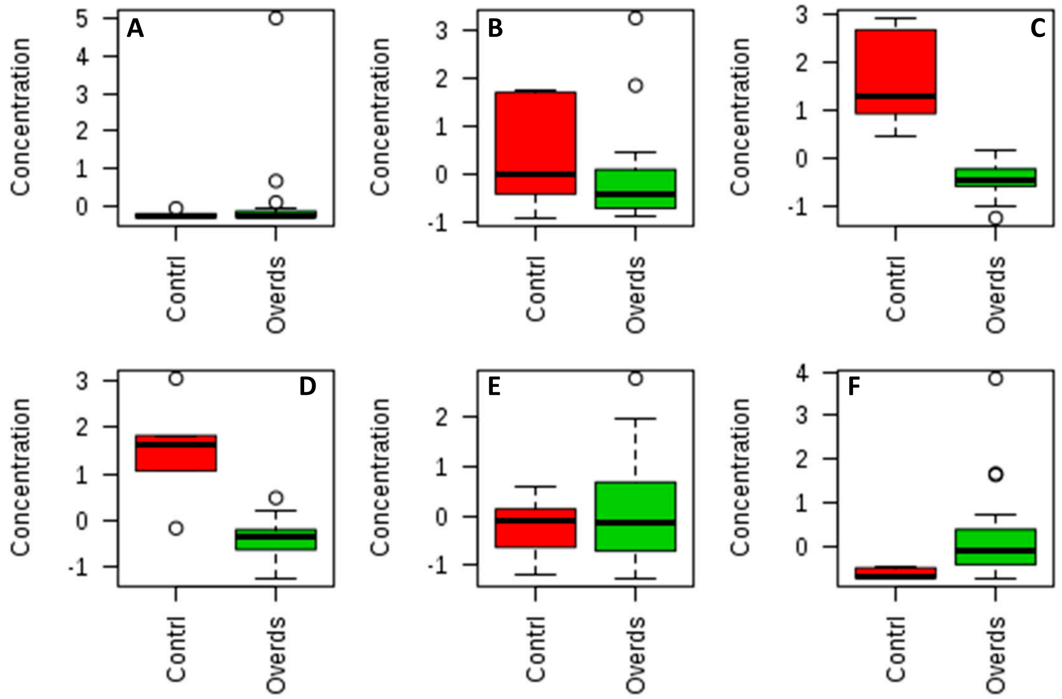

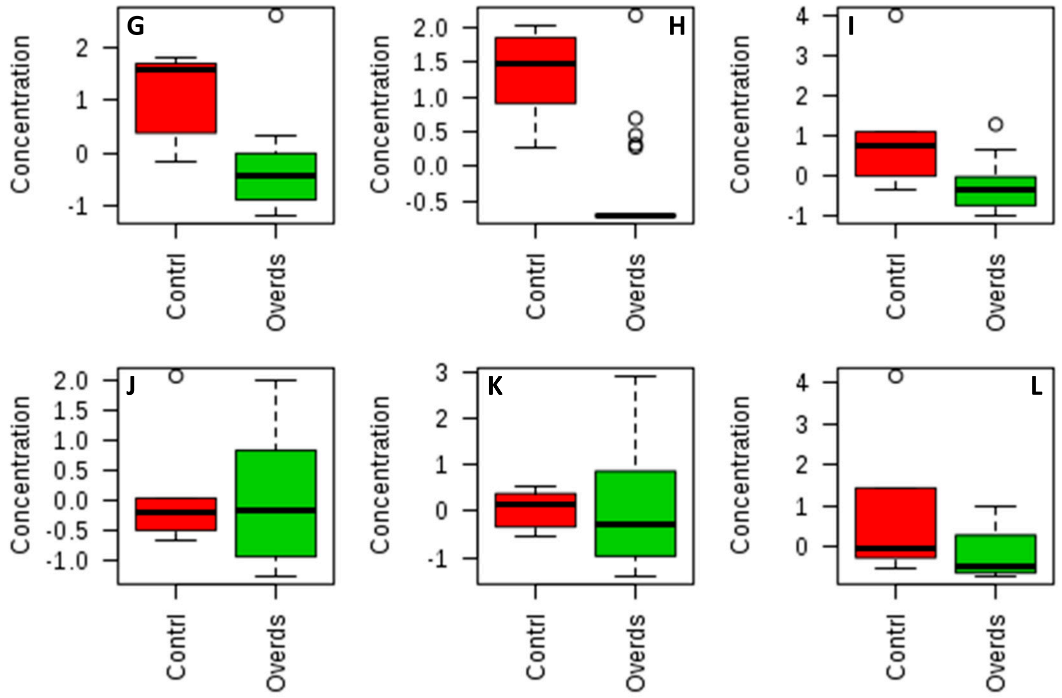


**Figure S16.** Pathway Views for a) propylene glycol, b) guanidoacetic acid, c) pyridoxamine, d) dihydrouracil, e) cis-acotinic acid, f) betaine, g) dihydrothymine, h) deoxycytidine, i) pantothenic acid, j) 4-pyridoxic acid, k) creatinine, and l) indoleacetic acid.

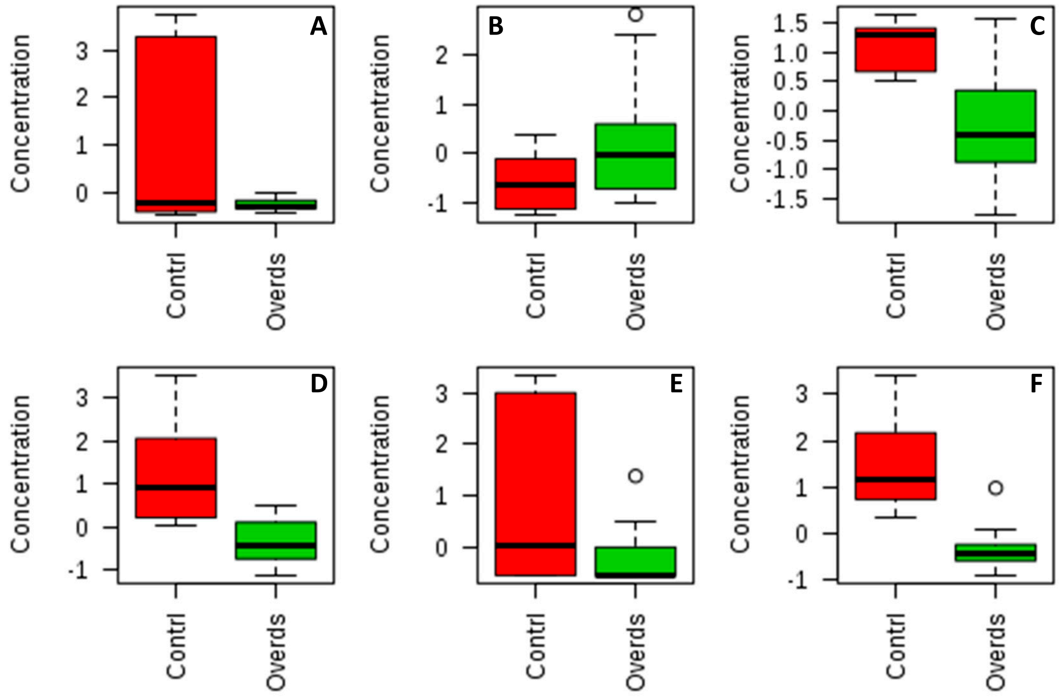

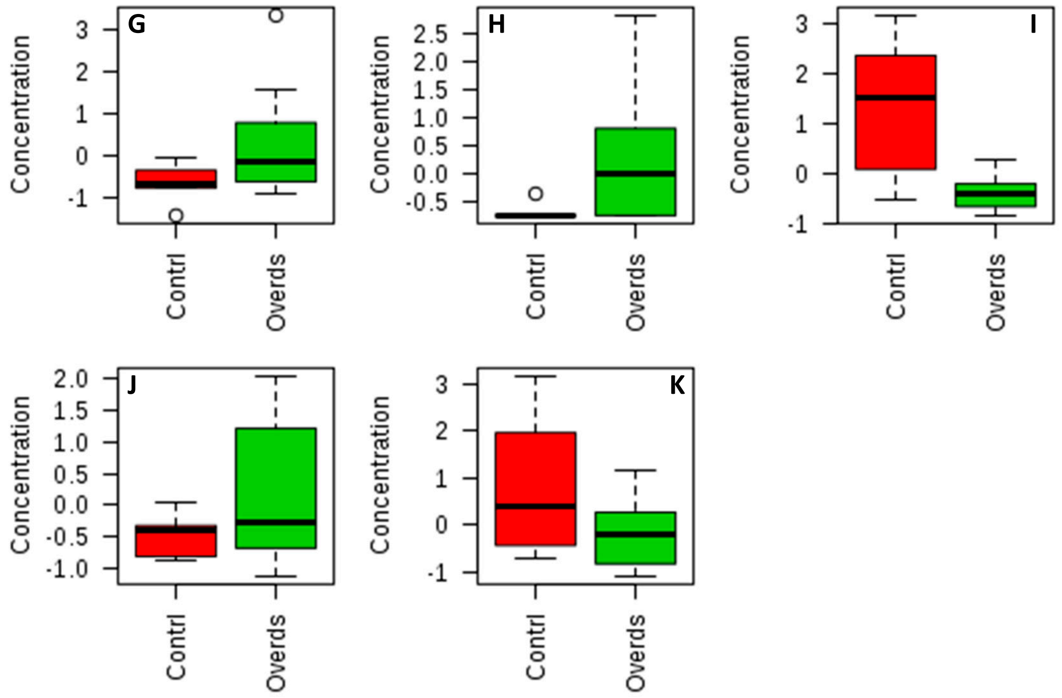


**Figure S17.** Pathway Views for a) phosphocreatine, b) indoleacetic acid, c) pyroglutamic acid, d) kynurenic acid, e) 4-hydroxyproline, f) xanthurenic acid, g) indoxyl, h) taurocholic acid, i) 2-oxoarginine, j) N-methylhydantoin, and k) 3-methylindoxyindole.

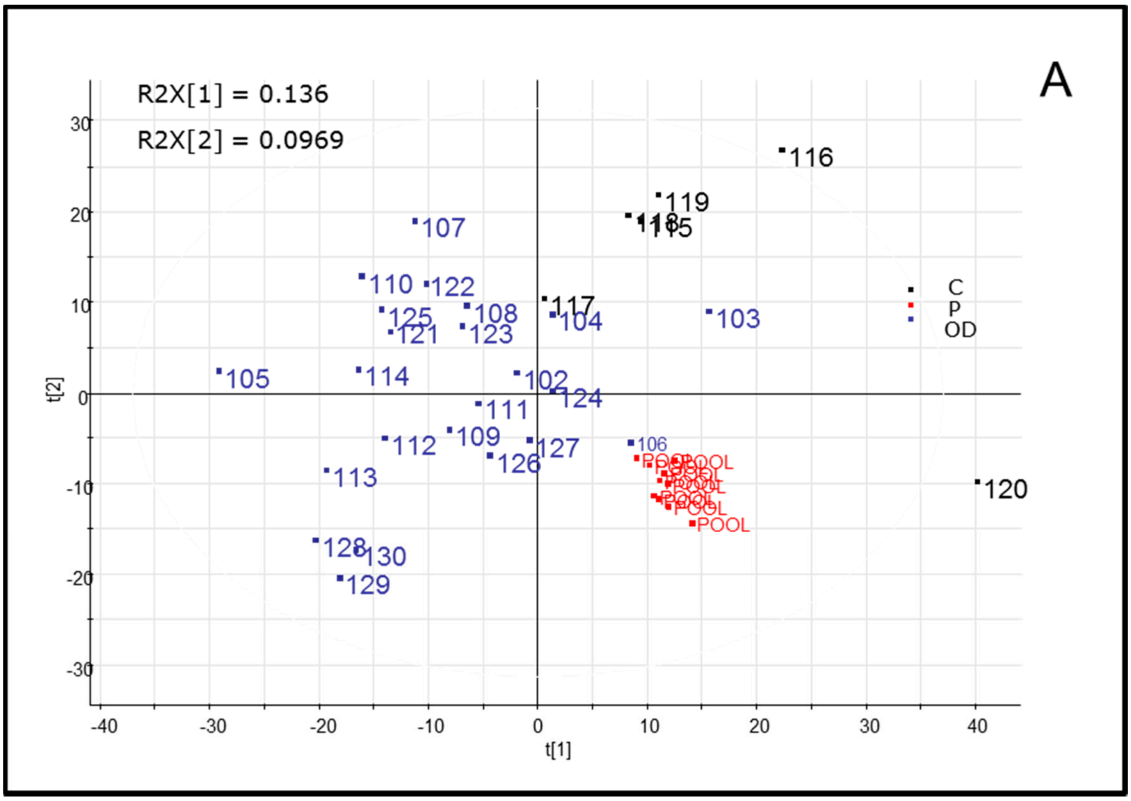

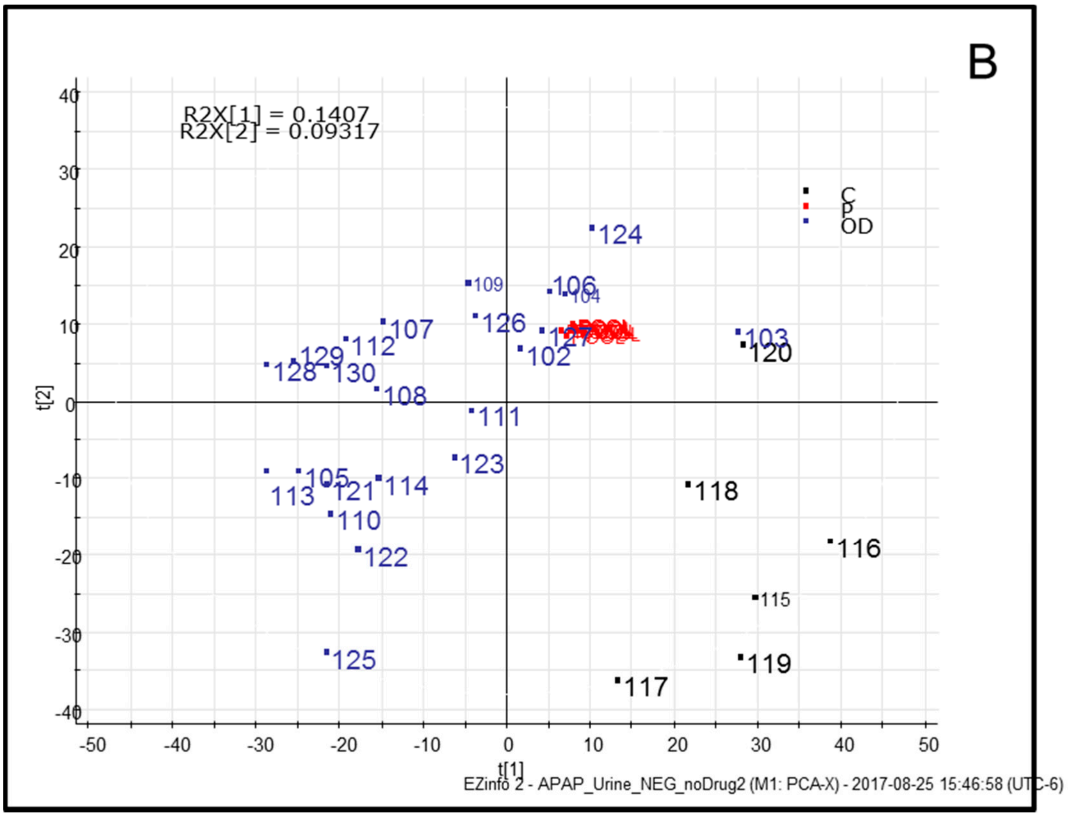


**Figure S18.** Principal component analysis (PCA) scores plots for both (A) positive and (B) negative ionization modes. Control samples are shown in black, samples from overdose subjects are in blue, and control pooled samples are in red.

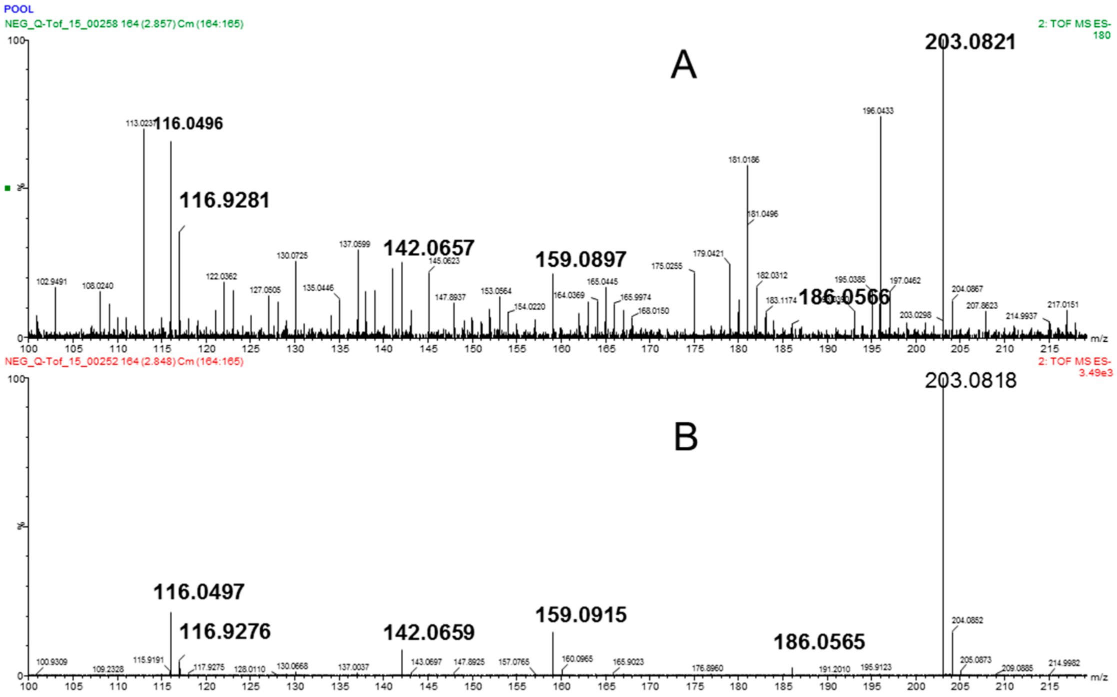


**Figure S19.** Simplified procedure for identification of an unknown ion at m/z 203.0821 in a sample; compare the fragmentation mass spectrum (A) with that from the standard spectrum of tryptophan (B). Retention time of the ion 203.0821 in the sample was 2.86 min, which was the same as the standard tryptophan, and the fragmentation mass spectrum (A) of the ion 203.0821 contains all of the fragments of tryptophan (B) with good mass accuracy (<6ppm).
